# Genome sequence and description of the heavy metal tolerant bacterium *Lysinibacillus sphaericus* strain OT4b.31

**DOI:** 10.4056/sigs.4227894

**Published:** 2013-10-02

**Authors:** Tito David Peña-Montenegro, Jenny Dussán

**Affiliations:** Centro de Investigaciones Microbiológicas – CIMIC, Universidad de los Andes, Bogotá, Colombia.

**Keywords:** *Lysinibacillus sphaericus* OT4b.31, DNA homology, *de novo* assembly, heavy metal tolerance, Sip1A coleopteran toxin

## Abstract

*Lysinibacillus sphaericus* strain OT4b.31 is a native Colombian strain having no larvicidal activity against *Culex quinquefasciatus* and is widely applied in the bioremediation of heavy-metal polluted environments. Strain OT4b.31 was placed between DNA homology groups III and IV. By gap-filling and alignment steps, we propose a 4,096,672 bp chromosomal scaffold. The whole genome (consisting of 4,856,302 bp long, 94 contigs and 4,846 predicted protein-coding sequences) revealed differences in comparison to the *L. sphaericus* C3-41 genome, such as syntenial relationships, prophages and putative mosquitocidal toxins. Sphaericolysin B354, the coleopteran toxin Sip1A and heavy metal resistance clusters from *nik, ars, czc, cop, chr, czr* and *cad* operons were identified. *Lysinibacillus sphaericus* OT4b.31 has applications not only in bioremediation efforts, but also in the biological control of agricultural pests.

## Introduction

Biological control of vector-borne diseases, such as dengue and malaria, and agricultural pests have been an issue of special concern in the recent years. Since Kellen et al. [1] first described *Lysinibacillus sphaericus* as an insect pathogen, studies have shown mosquitoes to be the major target of this bacterium [2-4], but toxic activity against other species has also been reported [5,6]. *L. sphaericus* larvicidal toxicity has been reported due to vegetative mosquitocidal toxins (Mtx) [7], the binary toxin (BinA/BinB) [4], Cry48/Cry49 toxin [8] and recently the S-layer protein [9]. To date, no larvicidal activity has been identified in *Lysinibacillus sphaericus* OT4b.31 against *Culex quinquefasciatus* [10].

On the other hand, *Lysinibacillus* species are potential candidates for heavy metal bioremediation. Some *Bacillaceae* strains have been successfully isolated from nickel contaminated soil [11], industrial landfills [12], naturally metalliferous soils [13] and a uranium-mining waste pile [14]. In addition, native Colombian *Lysinibacillus* strains have been reported as potential metal bioremediators. Strain CBAM5 is resistant to arsenic, up to 200 mM, and contains the arsenate reductase gene [15]. *L. sphaericus* OT4b.31 showed heavy metal biosorption in living and dead biomass. The S-layer protein was also shown to be present [16]. We observed 19 mosquito-pathogenic *L. sphaericus* strains and 6 non-pathogenic strains (including OT4b.31) that were able to grow in arsenate, hexavalent chromium and/or lead [17]. The moderate heavy metal tolerance in a *Lysinibacillus* strain isolated from a non-polluted environment generates interest in characterizing the genomic properties of *L. sphaericus* OT4b.31, in addition to its biotechnological potential in biological control.

Here we present a summary classification and a set of features for *Lysinibacillus sphaericus* OT4b.31 including previously unreported aspects of its phenotype, together with the description of the complete genomic sequencing and annotation.

## Classification and features

Formerly known as *Bacillus sphaericus,* the species was defined as having a spherical terminal spore and by its inability to ferment sugars [18]. According to physiological and phylogenetic analysis, it was reassigned to the genus *Lysinibacillus* [19]. Strains of *L. sphaericus* can be divided into five DNA homology groups (I–V). Some mosquito pathogenic strains are allocated in subgroup II-A, while *Lysinibacillus fusiformis* species is in subgroup II-B [20]. Later, according to 16S rDNA and lipid profile comparisons, *Lysinibacillus sphaericus* *sensu lato* was classified into seven similarity subgroups, of which only four retained the previous description by Krych et al. [21]. Recently, by using 16S rDNA phylogenetic analysis some mosquito pathogenic native strains were found in group II with heterogeneous heavy metal tolerance levels. [17].

**Figure 1:**
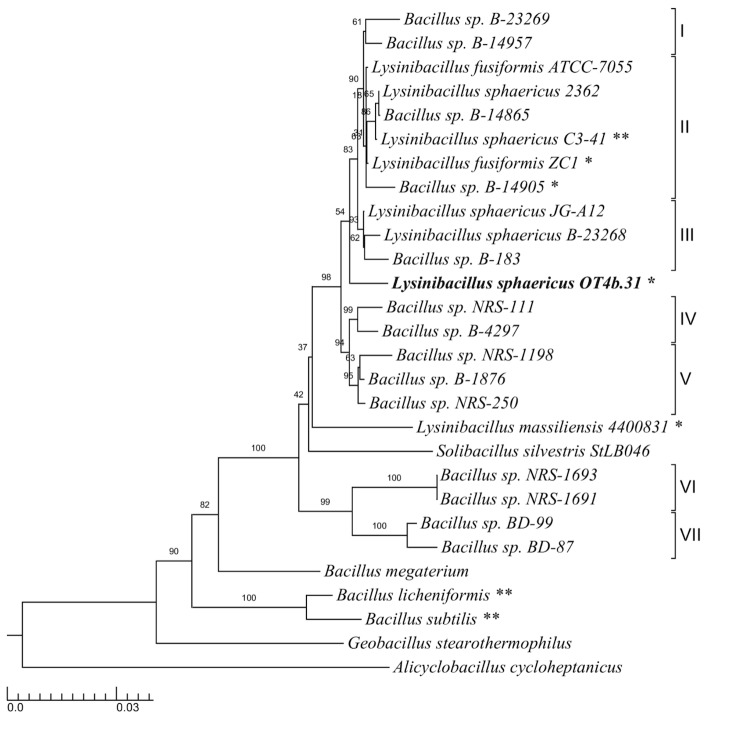
Phylogenetic tree highlighting the position of *Lysinibacillus sphaericus* OT4b.31 relative to the available type strains and other non-assigned species within the families *Alicyclobacillaceae* and *Bacillaceae*. *Alicyclobacillus cycloheptanicus* was designated as the outgroup species for the analyses. Right brackets encompass each homology group (I–VII) according to Nakamura’s benchmarks [21]. Nucleotide sequences obtained from GenBank and used in the phylogenetic analyses were as follows: *Alicyclobacillus cycloheptanicus* 1457 (X51928), *Geobacillus stearothermophilus* 10 (X57309), *Bacillus subtilis* 168^T^ (X60646), *Bacillus licheniformis* DSM 13^T^ (X68416), *Bacillus megaterium* IAM 13418^T^ (D16273), *Bacillus sp.* BD-87 (AF169520), *Bacillus sp.* BD-99 (AF169525), *Bacillus sp.* NRS-1691 (AF169531), *Bacillus sp.* NRS-1693 (AF169533), *Solibacillus silvestris* StLB046 (NR_074954), *Lysinibacillus massiliensis* 4400831 (NR_043092), *Bacillus sp.* NRS-250 (AF169536), *Bacillus sp.* B-1876 (AF169494), *Bacillus sp.* NRS-1198 (AF169528), *Bacillus sp.* B-4297 (AF169507), *Bacillus sp.* NRS-111 (AF169526), *Lysinibacillus sphaericus* OT4b.31 (AQPX00000042.1:91-1546), *Bacillus sp.* B-183 (AF169493), *Lysinibacillus sphaericus* B-23268^T^ (AF169495), *Lysinibacillus sphaericus* JG-A12 (AM292655), *Bacillus sp.* B-14905 (AF169491), *Lysinibacillus sphaericus* ZC1 (NZ_ADJR01000054.1:1-1487), *Lysinibacillus sphaericus* C3-41 (NC_010382.1:16887-18287), *Bacillus sp.* B-14865 (AF169490), *Lysinibacillus sphaericus* 2362 (L14011), *Lysinibacillus fusiformis* ATCC-7055 (AJ310083), *Bacillus sp.* B-14957 (AF169492) and *Bacillus sp.* B-23269 (AF169496). The branches are scaled in terms of the expected number of substitutions per site. Numbers adjacent to the branches represent percentage bootstrap values based on 1,000 replicates. Lineages with type strain genome sequencing projects registered in GOLD [22] are labeled with one asterisk, those also listed as 'Complete and Published' with two asterisks.

Partial 16S rRNA gene sequences (1,421 bp) were aligned to establish the phylogenetic neighborhood of *Lysinibacillus sphaericus* OT4b.31 (Figure 1). The phylogenetic tree was constructed by neighbor-joining [23] using the SEAVIEW [24] and TreeGraph2 [25] packages. Genetic distances were estimated by using the Jukes-Cantor model [23]. The stability of relationships was assessed by bootstrap analysis based on 1,000 resamplings for the tree topology. Interestingly, *L. sphaericus* OT4b.31 did not fall into any existing DNA similarity group; it was found between DNA similarity groups III and IV [21]. Consistent with Lozano & Dussán [17], *L. sphaericus* OT4b.31 did not fall into DNA similarity groups I, II or III.

**Figure 2:**
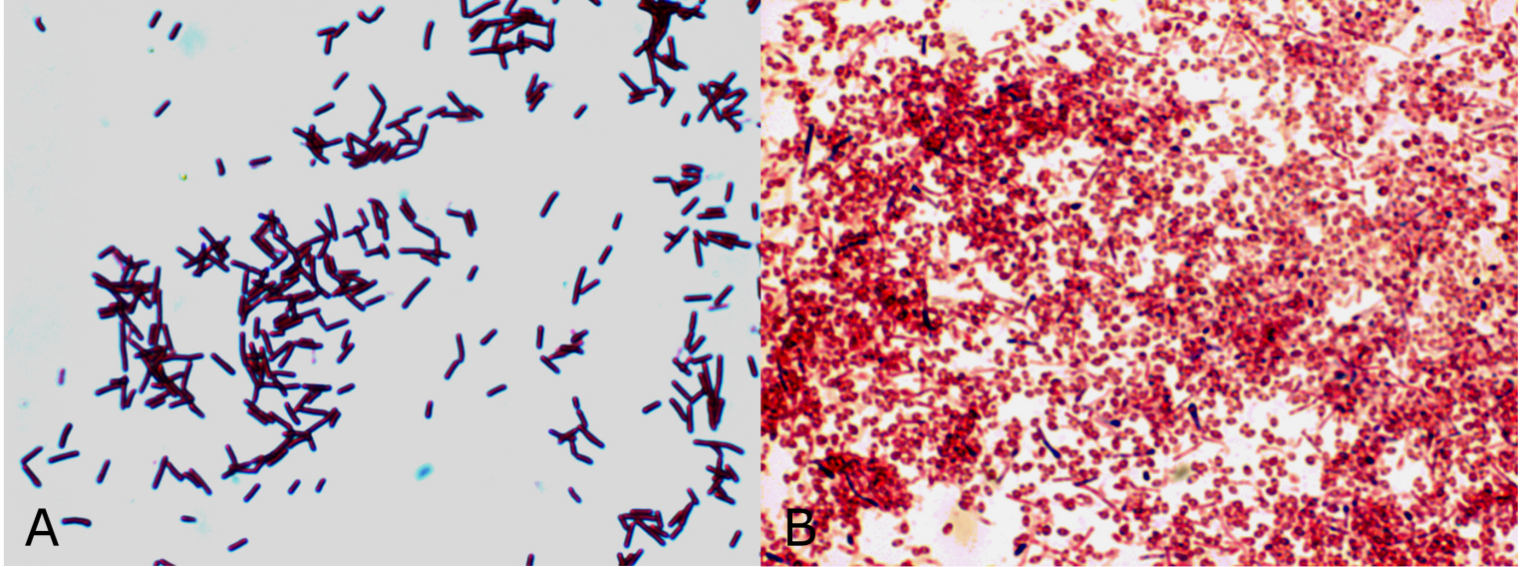
Gram staining of (A) vegetative cells and (B) spores of *Lysinibacillus sphaericus* OT4b.31.

**Figure 3:**
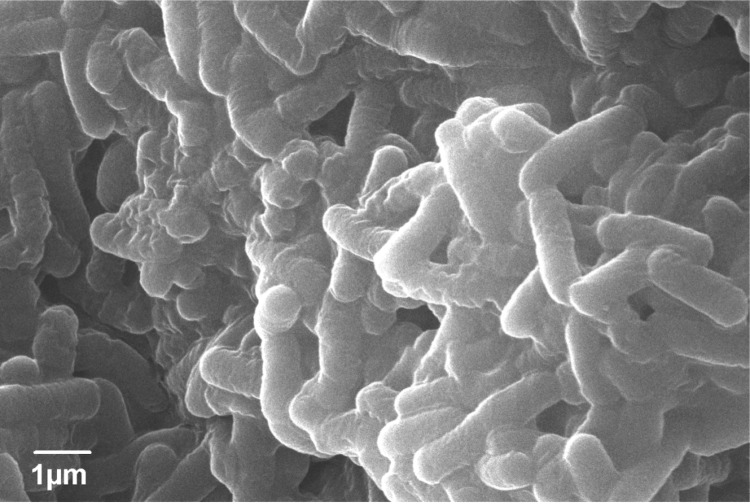
Scanning electron micrograph of *Lysinibacillus sphaericus* OT4b.31 at an operating voltage of 20 kV.

Dussán et al. [10] evaluated physiological diversity and genetic potential in native *Bacillaceae* isolates from highlands of the Colombian Andes, where *Lysinibacillus sphaericus* OT4b.31 was first described (Table 1). *L. sphaericus* OT4b.31 is an aerobic free-living bacterium isolated from coleopteran (beetle) larvae collected in the highlands of the Colombian Andes [10]. Vegetative cells stain Gram positive, but in sporulating stages, cell stain Gram variable (Figure 2). By using a JEOL JSM-5800LV (Japan) scanning electron microscope, *L. sphaericus* OT4b.31 is estimated to measure 0.61 to 0.65 µm in width and 1.9 to 2.3 µm long (Figure 3). *L. sphaericus* OT4b.31 showed slow sporulation rates (undetectable up to 40 hours of growth) and positive evidence of binary toxin which does not exhibit larvicidal activity against *Culex quinquefasciatus* [10]. Cultures grow at 10 to 40°C over a pH range of 6.0 to 9.0. Antibiotic resistance was evaluated separately by adding filter sterilized antibiotic solutions in Luria-Bertani broths and checking turbidity after 15 hours of growth. *L. sphaericus* OT4b.31 is sensitive to kanamycin (12.5 µg/mL), chloramphenicol (25 µg/mL), erythromycin (5 µg/mL), and gentamicin (25 µg/mL), while it showed resistance to trimethoprim/sulfamethoxazol up to 30 µg/mL/150 µg/mL.

**Table 1:** Classification and general features of *Lysinibacillus sphaericus* OT4b.31 according to the MIGS recommendations [26]

**MIGS ID**	**Property**	**Term**	**Evidence code**^a^
	Current classification	Domain *Bacteria* Phylum *Firmicutes* Class *Bacilli* Order *Bacillales* Family *Bacillaceae* Genus *Lysinibacillus* Species *Lysinibacillus sphaericus* Type strain OT4b.31	TAS [27] TAS [28-30] TAS [31,32] TAS [33,34] TAS [33,35] TAS [19,36] TAS [19,37] TAS [10]
	Gram stain	Positive in vegetative cells, variable in sporulating stages	IDA
	Cell shape	Straight rods	IDA
	Motility	Non-motile	IDA
	Sporulation	Sporulating	IDA
	Temperature range	Mesophile, grows > 14°, < 37°C	TAS [10]
	Optimum temperature	30°C	TAS [10]
	Carbon source	Complex carbohydrates	TAS [10]
	Energy metabolism	Heterotroph	TAS [10]
MIGS-6	Habitat	Coleopteran (beetle) larvae	TAS [10]
MIGS-6.3	Salinity	Growth in Luria-Bertani broth (5% NaCl)	IDA
MIGS-22	Oxygen requirement	Aerobic	TAS [10]
MIGS-15	Biotic relationship	Free living	TAS [10]
MIGS-14	Pathogenicity	Unknown	TAS [10]
MIGS-4	Geographic location	Tenjo, Cundinamarca, Colombia	TAS [10]
MIGS-5	Sample collection time	1995	TAS [10]
MIGS-4.1	Latitude	4.88727	TAS [10]
MIGS-4.2	Longitude	-74.132831	TAS [10]
MIGS-4.3	Depth	Surface	TAS [10]
MIGS-4.4	Altitude	2,685 m above sea level	TAS [10]

a) Evidence codes - IDA: Inferred from Direct Assay; TAS: Traceable Author Statement (i.e., a direct report exists in the literature); NAS: Non-traceable Author Statement (i.e., not directly observed for the living, isolated sample, but based on a generally accepted property for the species, or anecdotal evidence). These evidence codes are from the Gene Ontology project [38].

## Genome sequencing information

### Genome project history

The genome sequencing of *Lysinibacillus sphaericus* OT4b.31 was supported by the CIMIC (Centro de Investigaciones Microbiológicas) laboratory at the University of Los Andes within the Grant (1204-452-21129) of the Instituto Colombiano para el fomento de la Investigación Francisco José de Caldas. Whole genomic DNA extraction and bioinformatics analysis was performed at CIMIC laboratory, whereas libraries construction and whole shotgun sequencing at the Beijing Genome Institute (BGI) Americas Laboratory (Tai Po, Hong Kong). The applied pipeline included quality check of reads, de novo assembly, a gap-filling step and mapping against a reference genome. This whole genome shotgun project has been deposited at DDBJ/EMBL/GenBank under the accession AQPX00000000. The version described in this paper is the first version, AQPX01000000. A summary of the project information is shown in Table 2.

**Table 2:** Genome sequencing project information

**MIGS ID**	**Property**	**Term**
MIGS-31	Finishing quality	Improved high-quality draft
MIGS-28	Libraries used	One paired end tags 90:90 bp with 500 bp insert
MIGS-29	Sequencing platforms	Illumina Hi-Seq 2000
MIGS-31.2	Fold coverage	100×
MIGS-30	Assemblers	CLC Assembly Cell version 4.0.10
MIGS-32	Gene calling method	Glimmer3, tRNAscan-SE
	Genbank ID	AQPX00000000
	Genbank Date of Release	May 10, 2013
	GOLD ID	Gi39289
	Project relevance	Biotechnology, metabolic pathway

### Growth conditions and DNA isolation

*Lysinibacillus sphaericus* strain OT4b.31 was grown in nutrient broth for 16 hours at 30ºC and 150 rev/min. High molecular weight DNA was isolated using the EasyDNA^®^ Kit (Carlsbad, CA, USA. Invitrogen) as indicated by the manufacturer. DNA purity and concentration were determined in a NanoDrop spectrophotometer (Wilmington, DE, USA. Thermo Scientific).

### Genome sequencing and assembly

After DNA extraction, samples were sent to the Beijing Genome Institute (BGI) Americas Laboratory (Tai Po, Hong Kong). Purified DNA sample was first sheared into smaller fragments with a desired size by a Covaris E210 ultrasonicator. Then the overhangs resulting from fragmentation were converted into blunt ends by using T4 DNA polymerase, Klenow Fragment and T4 polynucleotide kinase. After adding an “A” base to the 3' end of the blunt phosphorylated DNA fragments, adapters were ligated to the ends of the DNA fragments. The desired fragments were purified though gel-electrophoresis, then selectively enriched and amplified by PCR. The index tag was introduced into the adapter at the PCR stage as appropriate, and a library quality test was performed. Lastly, qualified, short, paired-ends of 90:90 bp length with 500 bp insert libraries were used to cluster preparation and to conduct whole-shotgun sequencing in Illumina Hi-Seq 2000 sequencers.

Using the FASTX-Toolkit version 0.6.1 [39] and clean_reads version 0.2.3 from the ngs_backbone pipeline [40] reads were trimmed and quality filtered. Then, with the CLC Assembly Cell version 4.0.10 [41], assembly and scaffolding steps were conducted via a de novo assembly pipeline. The assembly included automatic scaﬀolding and k-mer/overlapping optimization steps. Some gaps were successfully filled by using GapFiller [42] within 30 iterations. No more gaps reached convergence by running more iterations. To obtain structural insight of a chromosomal scaffold, we used CONTIGuator.2 [43], using the *Lysinibacillus sphaericus* strain C3-41 chromosome (accession number: CP000817.1) as reference. Gap-filling steps and mapping to reference sequences were performed again to confirm convergence. Quality assessment of the assembly was performed with iCORN [44]. The error rate of the final assembly is less than 1 in 1,000,000. Lastly, by using PROmer from the MUMmer [45] and Mauve [46] packages, we compared the chromosomal assembly and the chromosome of *L. sphaericus* C3-41.

### Genome annotation

The Glimmer 3 gene finder was used to identify and extract sequences for potential coding regions. To achieve the functional annotation steps, the RAST server [47] and Blast2GO pipelines [48] were used. Blast2GO performed the blasting, GO-mapping and annotation steps; which included a description according to the ProDom, FingerPRINTScan, PIR-PSD, Pfam, TIRGfam, PROSITE, ProDom, SMART, SuperFamily, Pattern, Gene3D, PANTHER, SignalIP and TM-HMM databases. The results were summarized with InterPro [49]. Additionally, a GO-EnzymeCode mapping step was used to retrieve KEGG pathway-maps. tRNA genes were identified by using tRNAscan-SE [50] and rRNA genes by using RNAmmer [51]. The possible orthologs of the genome were identified based on the COG database and classified accordingly [52]. Prophage region prediction was also conducted by using the PHAST tool [53].

## Genome properties

**Figure 4:**
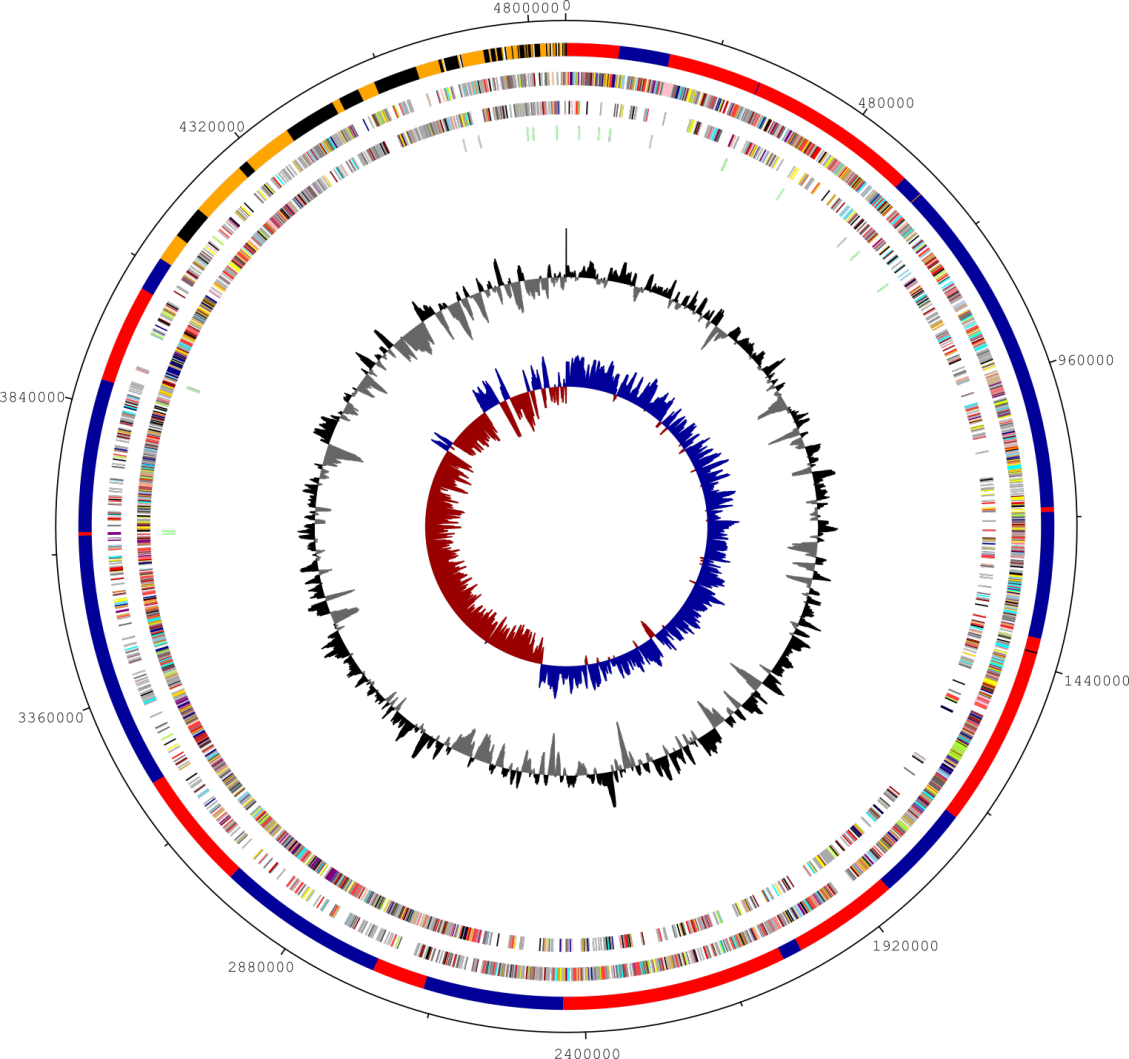
Graphical map of the genome. From outside to the center: Ordered and oriented scaffolds assigned to chromosome in blue and red, extrachromosomal scaffolds in orange and black, Genes on forward strand (color by COG categories), Genes on reverse strand (color by COG categories), RNA genes (tRNAs green, rRNAs gray), GC content and GC skew.

The genome summary and statistics are provided in Tables 3 and 4 and Figure 4. The genome consists of 96 scaffolds in 4,856,302 bp total size with a GC content of 37.5%. A total of 23 scaffolds were successfully aligned to a reference sequence, comprising 4,096,672 bp of sequence and are represented by the red and blue bars within the outer ring of Figure 4. Of the 4,938 genes predicted, 4,846 were protein-coding genes, 46 RNAs, and 1,623 pseudogenes were identified. Genes assigned a putative function comprised 67.13% of the protein-coding genes while the remaining ones were annotated as hypothetical proteins. The distribution of genes into COGs functional categories is presented in Table 5.

**Table 3:** Summary of genome

**Label**	**Size (Mb)**	**Topology**	**INSDC identifier**
Chromosomal scaffold	4,096,672	Circular	KB933398.1
Extrachromosomal elements	759,630	Linear	KB933399.1-KB933469.1

**Table 4:** Nucleotide content and gene count levels of the genome

**Attribute**	Value	% of total^a^
Genome size (bp)	4,856,302	100.00
DNA GC content (bp)	1,821,262	37.50
DNA coding region (bp)	3,924,297	80.81
Number of replicons	1	
Extrachromosomal	0	
Total genes	4,938	100
RNA genes	46	0.93
rRNA operons	7	
tRNA genes	38	0.77
Pseudogenes	1,623	32.87
Protein-coding genes	4,846	98.14
Genes in paralog clusters	658	13.33
Genes assigned to COGs	2,946	59.66
1 or more conserved domains	2,946	59.66
2 or more conserved domains	529	10.71
3 or more conserved domains	98	1.98
Genes with function prediction	3,315	67.13
Genes assigned Pfam domains	2,799	56.68
Genes with signal peptides	1,206	24.42
Genes with transmembrane helices	1,206	24.42
CRISPR repeats	0	0.00

a) The total is based on either the size of the genome in base pairs or the total number of protein coding genes in the annotated genome.

**Table 5:** Number of genes associated with the 25 general COG functional categories

**Code**	**Value**	**%age**^a^	**Description**
J	180	3.80	Translation
A	118	2.49	RNA processing and modification
K	354	7.48	Transcription
L	167	3.53	Replication, recombination and repair
B	1	0.02	Chromatin structure and dynamics
D	37	0.78	Cell cycle control, mitosis and meiosis
Y	0	0	Nuclear structure
V	75	1.58	Defense mechanisms
T	293	6.19	Signal transduction mechanisms
M	159	3.36	Cell wall/membrane biogenesis
N	95	2.01	Cell motility
Z	31	0.66	Cytoskeleton
W	28	0.59	Extracellular structures
U	48	1.01	Intracellular trafficking and secretion
O	96	2.03	Posttranslational modification, protein turnover, chaperones
C	169	3.57	Energy production and conversion
G	146	3.09	Carbohydrate transport and metabolism
E	351	7.42	Amino acid transport and metabolism
F	85	1.80	Nucleotide transport and metabolism
H	142	3.00	Coenzyme transport and metabolism
I	133	2.81	Lipid transport and metabolism
P	273	5.77	Inorganic ion transport and metabolism
Q	98	2.07	Secondary metabolites biosynthesis, transport and catabolism
R	450	9.51	General function prediction only
S	234	4.95	Function unknown
-	1,694	37.74	Not in COGs

a) The total is based on the total number of protein coding genes in the annotated genome.

## Insights into the genome

**Figure 5:**
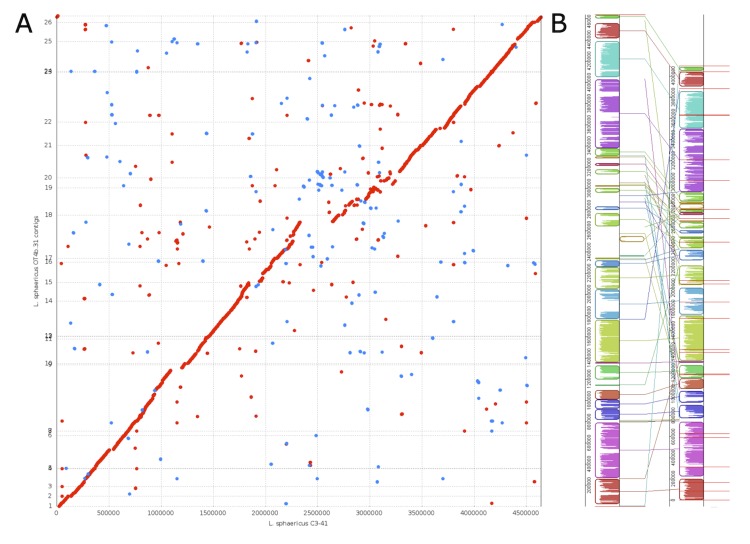
(A) Dot-plot of amino-acid-based alignment of a 4.09 Mbp chromosomal scaffold of *L. sphaericus* OT4b.31 (y-axis) to a 4.6 Mbp chromosome of *L. sphaericus* C3-41 (x-axis). Aligned segments are represented as dots or lines. Forward matches are plotted in red, reverse matches in blue. Figure generated by PROmer [45]. (B) Nucleotide-based alignment of a 4.09 Mbp chromosomal scaffold of *L. sphaericus* OT4b.31 (right) to a 4.6 Mbp chromosome of *L. sphaericus* C3-41 (left). A total of 27 homologous blocks are shown as identically colored regions and linked across the sequences. Regions that are inverted relative to *L. sphaericus* OT4b.31 are shifted to the right of center axis of the sequence. The origin of replication in each sequence is approximately at coordinate 1. Red bars show the limits of each contig in the chromosomal scaffold. Contigs 1 to 26 are numbered in ascending order start in coordinate 1. The figure was generated by Mauve [46].

To complete the assembly process, a resequencing pipeline was applied that set whole genome sequences as references such as *Lysinibacillus sphaericus* C3-41, *Bacillus sp.* strain B-14905, *Bacillus sp.* NRRL B-14911, *Bacillus megaterium* QM B1551, *Bacillus anthracis* Ames, *Lysinibacillus boronitolerans* F1182 and *Lysinibacillus fusiformis* ZC1. Mapping coverage was lower than 30% in any case (data not shown). In addition, GC content, and depth–GC correlation analysis demonstrated neither a biased distribution nor heterogeneity in the GC content of raw data. Thus, a *de novo* assembly was conducted in the CLC Assembly Cell version 4.0.10, as discussed above, resulting in a 123-scaffold assembly with a N50=96,816 bp. After the gap-filling step, all intrascaffold gaps and 29 interscaffold gaps were closed, leaving 94 scaffolds with a N50=205,086 bp. Finally, a mapping step was conducted using the sequences mentioned above as references. This yielded 26 supercontigs that mapped to *L. sphaericus* strain C3-41 chromosome corresponding to 88.9% of the reference chromosome. This alignment was proposed as a chromosomal scaffold. Other reference sequences lead to no significant coverage levels and extrachromosomal scaffolds did not align to previously sequenced plasmids of related species (data not shown). Chromosomal comparison from the PROmer analysis between *L. sphaericus* strains OT4b.31 and C3-41 showed that most of the two chromosomes mapped onto each other, revealing large segments of high similarity (Figure 5). However, a region comprising around 2 to 3.25 Mbp in the C3-41 chromosome and the contigs 15 to 19 in the chromosomal scaffold were remarkably scattered in the dot-plot, revealing low coverage levels and different syntenial relationships to the reference sequence.

The origin of replication of the chromosome of *L. sphaericus* OT4b.31 was estimated by similarities to several features of the corresponding regions in *L. sphaericus* C3-41, *Bacillus sp.* B-14905 and other close related bacteria, including colocalization of the genes: *dnaX*, *recR*, *holB, dnaA*, *recG* and *recA*; and GC nucleotide skew [(G–C)/(G+C)] analysis. In the first 40 Kbp of contig 1, we found *dnaX, recR,* and *holB,* while *dnaA, recG* and *recA* were found at the end (after 290 Kbp) of contig 13. This may suggest that contig 13 should be allocated immediately before contig 1. Besides, there was no evidence of multiple *dnaA* boxes around the potential origin. The replication termination site of the chromosomal scaffold is believed to be localized near 2.5 Mbp in the contig 18, according to GC skew analysis, and the coding bias for the two strands of the chromosome is for the majority of CDSs to be on the outer strand from 0 to ~2.5 Mbp and on the inner strand from ~2.5 Mbp to the end of the chromosomal scaffold (contig 26, Figure 4). This was also confirmed by the presence of *parC* (H131_12178) and *parE* (H131_12183), which encode the subunits of the chromosome-partitioning enzyme topoisomerase IV [54]. Similar to the *L. sphaericus* C3-41 genome [55], we did not find the homolog of *rtp* (replication terminator protein-encoding gene) in the chromosomal assembly of OT4b.31.

A total of 42 hypothetical protein coding sequences were assigned as putative transposable elements, with the most frequent families being IS66, IS110, IS1272 and IS3. In addition, five prophage regions were identified, of which one region is intact and 4 regions are incomplete. *Lactobacillus* phage C5 (intact), *Bacillus* phage φ105, *Clostridium* phage c-st, *Bacillus* Phage SPP1 and *Bacillus* phage Wβ predicted regions were allocated at contigs 34, 8, 15, 18 and 37, respectively. Only lysis proteins were predicted in phages C5 and c-st regions. The only genes remaining in the phage φ105 region are those for coat proteins, integrase, and hypothetical and phage-like coding sequences. This is probably the remnant of phage invasion and genome deterioration during evolution. In addition, any previously reported phages in the genome of *L. sphaericus* C3-41 are in the genome of OT4b.31.

Two elements contain conserved domains from the *Listeria* pathogenicity island LIPI-1, functionally assigned as a thiol-activated cytolysin and a phosphatidylinositol phospholipase C. The first was confirmed to correspond to the *L. sphaericus* B354 sphaericolysin coding gene in contig 18 (H131_12483). Sphaericolysin B354 has been reported to be widespread across *L. sphaericus* DNA homology groups not only including IIA, IIB, IV and V [56] but also non-grouped species such as OT4b.31. Upstream, in the same contig, a *Bacillus* toxin from the family Mtx2 (PFam PF03318) was found and described as a hypothetical Sip1A toxin coding sequence (H131_12498). Purified from *Bacillus thuringiensis* strain EG2158, Sip1A is a secreted insecticidal protein of 38 KDa having activity against Colorado Potato beetle (*Leptinotarsa decemlineata*) [57]. Considering that *L. sphaericus* OT4b.31 was isolated from beetle larvae, we suggest potential coleopteran larvicidal activity. To our knowledge, strain OT4b.31 is the first report of a predicted Sip1A-like toxin in a native *Lysinibacillus sphaericus*. Unexpectedly, *mtx* or *bin* mosquito pathogenic genes were not found in the OT4b.31 genome, despite a previous report showing positive evidence of BinA/B toxins with no larvicidal activity [10].

A total of 32 CDSs were described as surface (S) layer proteins or S-layer homologs (SLH). The putative S-layer gene *sllB* (H131_05299) previously reported in *L. sphaericus* JG-A12 [58] was found in a 3,696 bp sequence allocated in contig 8. Three sequences with conserved domains similar to Slp5 and Slp6 were identified in contigs 8 (H131_05339, H131_05344) and 22 (H131_16838). *Bacillus sp.* B-14905 was the most similar sequence for the majority of S-layer protein domains. In addition, a putative glycoprotein (H131_22117), a bifunctional periplasmic precursor (H131_05993) and an S-layer fusion (H131_05409) coding sequence associated with S-layer proteins were recognized. On the other hand, a cluster of spore germination genes were determined near the termination of the replication site (including genes from the *ger* and *ype* operons) among other genes widespread in the genome. Three clusters of sporulation genes were allocated at contigs 1, 10 and 13 (including genes from *spo*II, *spo*V, *yaa* and *sig* operons).

Responses against toxic metal(oid)s in *L. sphaericus* OT4b.31 could be controlled by efflux pumps related genes in clusters found in contigs. Putative coding sequence order is as follows: *yozA*→*czcD*→*csoR*→*copZA* (contig 1, H131_00045: H131_00065); *nikABC*→*oppD*→*nikD* (contig 17, H131_11103:H131_11123); *cadC*-like→*cadA* (contig 24, H131_17086:H131_17081); *arsRBC* – putative extracellular secreted protein CDS – *arsR*-like→*arsR*-like→ putative excinuclease CDS (contig 18, H131_11998:H131_12028). The function of YozA is still unknown [59], but is similar to CzrA and CadC belonging to the ArsR transcriptional family regulators. YozA, CsoR (from the copper-sensitive operon), CadC-like and ArsR proteins seem to be the direct regulators of each cluster. At least one additional copy of ChrA, CzrB and CzcD CDSs were found. Upstream the *nik* cluster, we could not find transcriptional regulators. In summary, *L. sphaericus* OT4b.31 has protein encoding sequences probably involved in the resistance against Cd, Zn, Co, Cu, Ni, Cr, and As. In fact, prior reports of resistance to toxic metals [16,17] in *L. sphaericus* OT4b.31 may be explained due to participation of heavy-metal resistance proteins.

Strain OT4b.31 probably has a diverse defense repertoire according to the following responses and predicted genes: bacitracin stress responses, genes *bceBASR* and *yvcPQRS;* multidrug resistance, MATE (multidrug and toxin extrusion) family efflux pump genes *ydhE*/*norM* and *acrB*; antibiotics resistance, genes *vanRSW*, *tetP*-like group II, *fusA* (elongation factor G), *fosB*, *blaZ* and *ampC*-like. Based in the KEGG analysis, some predicted proteins might participate in peripheral pathways for the degradation of benzoate, aminobenzoate, quinate, toluene, naphthalene, geraniol, limonene, pinene, chloroalkane, chloroalkene, styrene, ethilbenzene, caprolactam and atrazine compounds, and biosynthesis of streptomycin, novobiocin, zeatin, ansamycins, penicillin and cephalosporins.

## Conclusions

The native Colombian strain *Lysinibacillus sphaericus* OT4b.31, isolated from beetle larvae, is classified between DNA similarity groups III and IV. A comparison of the chromosomal sequences of strain OT4b.31 and its closest complete genome sequence, *L. sphaericus* C3-41, demonstrates the presence of only a few similar regions with syntenial rearrangements, and no prophage or putative mosquitocidal toxins are shared. Sphaericolysin B354 and the coleopteran toxin Sip1A were predicted in the strain OT4b.31, a finding which may be useful not only in bioremediation of polluted environments, but also for biological control of agricultural pests. Finally, Cd, Zn, Co, Cu, Ni, Cr and As resistances probably are supported by efflux pumps genes.
